# Bone Mineral Density and Bone Metabolism in Patients with Duchenne Muscular Dystrophy 

**Published:** 2018

**Authors:** Mohammad BARZEGAR, Elnaz NIKNAM, Parinaz HABIBI, Shadi SHIVA, Sanaz TAHMASEBI

**Affiliations:** 1 Department of Neurology, Pediatric Health Research Center, Tabriz University of Medical Sciences, Tabriz, Iran.

**Keywords:** Duchene muscular dystrophy, Bone mineral density, Corticosteroid, Vitamin D, Osteoporosis

## Abstract

**Objective:**

Poor bone health with related morbidity is a major problem with Duchene Muscular Dystrophy (DMD). Decreased mobility and long-term corticosteroid therapy are involved in poor bone health in DMD. We investigated bone mineral density and bone metabolism in 30 steroid treated DMD patients and also compared mentioned factors between ambulated and non-ambulated patients.

**Materials & Methods:**

In this cross-sectional study, 30 boys (21 patients ambulate and 9 non-ambulate) with documented DMD, according to genetic analysis, were enrolled in 2015. Demographic characteristics, neurologic exam findings, muscle function score, corticosteroid dose and duration and food frequency questionnaire were recorded. Bone mineral density was measured with dual- energy X-ray absorptiometry (DEXA) on lumbar spine and left proximal femur. Serum 25-hydroxyvitamin D, calcium, phosphorus and parathyroid hormone (PTH) levels were measured.

**Results:**

Osteoporosis was found in 86.7% patients. Mean bone density in the lumbar spine was -1.5±0.24 and -1.4±0.27 in ambulates and non-ambulates respectively (*P*=0.7). Mean bone density at proximal femur was -3.4±0.2 in ambulates and -3.4±0.3 in non-ambulates (*P* =0.48).

Intra-groups statistical analysis showed significant difference between bone mineral density at lumbar spine and proximal femur in both mentioned groups (*P*<0.05). Vitamin D deficiency was detected in 13 patients (43.3%) and its serum level was significantly lower in non-ambulates compared with ambulates.

**Conclusion:**

Considering high prevalence of vitamin D deficiency and osteoporosis in DMD patients, it seems vitamin D supplementation can improve vitamin D status and osteoporosis in these patients, especially in non-ambulates.

## Introduction

 Duchene Muscular Dystrophy (DMD) is an X-linked recessive disease that affects 1 in 3500-6000 male births. This condition is resulting from mutations (mainly deletions) in the dystrophin gene located at Xp21.2. leading to either an absence of protein dystrophin or its defect causing progressive muscle degeneration. Historically patients with DMD are usually wheelchair-bound before the age from 12 yr and often die in their early 20s (1-3). Although no specific causal treatments for DMD have been established, corticosteroids have decreased the progression of the disease and improve muscular strength (4, 5). The natural history of the disease can be altered by targeting the interventions to known manifestations and complications such as respiratory, cardiac, musculoskeletal and psychological symptoms that can lead to improvement in function, quality of life and longevity in children currently being diagnosed with DMD and raising their life expectancy toward their fourth decade (6-8). 

 Poor bone health is a common feature of patients with DMD and the cause of significant morbidity including increased fracture rates, bone pain, osteopenia and osteoporosis. It might be because of any pathological changes; on the other hand love level of vitamin D and/or prolonged administration of corticosteroids can worsen this process (9, 10). Osteoporosis in children is defined by the presence of vertebral compression fractures without local disease or trauma, or the presence both a clinically significant fracture history and bone mineral density (BMD) greater or equal to 2 standard deviations (Z-score) below age and sex related values (11). For some patients with DMD the occurrence of a long bone fracture heralds the end of ambulation (12). Previous international courses were held in 2004 on the DMD patients and osteoporosis related problems. Experts in orthopedics, genetics, endocrinology, nutrition, and rehabilitation medicine were assembled in Birmingham, UK and Cincinnati, Ohio USA to review the knowledge about the prevalence and cause of bone fragility and to propose future directions for the treatment. An important meeting through the Atlantic area was held regarding on the importance of this disease and it related problems (7, 13).

 In Division of Pediatric Neurology of Tabriz Children Hospital, Tabriz, Iran steroids are part of the routine treatment of DMD care. We investigated the aspects of bone mineral density and bone metabolism in 30 male patients with DMD in North-West Iran to assure subsequent accurate interventions and treatments.

## Materials & Methods

 This cross-sectional study was conducted during January to May 2015. Thirty DMD patients (documented by genetic analysis) were enrolled. Demographic characteristics, neurologic exam findings, drug history and muscle function score were recorded. All of the patients were on long-term corticosteroid therapy. The muscle functional grading was classified according to Vignos scale (grades 1-9) (14). 

Informed consent was taken from the patients and the study was approved by the Ethics Committee of the university.

BMD was measured with dual- energy X-ray absorptiometry (DEXA; HOLOGIC, QDR 2000, EXPLORER S1N 90910) at lumbar spine and left proximal femur in the same referral center for all patients. Body weight and height were measured before DEXA scan. The DEXA results were reported in lumbar spine (L1, L2, L3 and total) and proximal of femur (neck, trochanter, intertroch and total). BMD greater or equal to 2 standard deviations (Z-score) below age and sex related values defined osteoporosis (15).

BMD is reported in grams per square centimeter (a two-dimensional measurement). Both bone mineral content and the geometry (macro-architecture) of the site measured. Serum calcium, phosphorus, alkaline phosphatase (calorimetric method, bionic kit, Hitachi 917), parathyroid hormone (ECL, Immulit 2000, normal range: 15-65) and serum 25-hydroxy vitamin D (ELISA, Algeria) level were checked for all patients. The severity of 25OH vitamin D deficiency was classified according to pre-defined standards (16).

 A food-frequency questionnaire (FFQ) was used to assess average intakes of 52 items of foods and beverages over the past year. All common food sources of calcium, phosphorus and vitamin D were included in FFQ.Three day food records were obtained additionally. Each food and beverage was then coded according to Iranian food items and analyzed for content of nutrients by nutritionist IV software (first Databank, Hearst Corp, San Bruno, Ca, USA) (17).

The study was not specifically aimed at evaluating the fracture rate in DMD children and only history of fractures was sought.

 Statistical analysis was performed using SPSS 17 (Chicago, IL, USA). Univariate analysis was used to determine factors associated with the development of osteoporosis in patients. Chi-square and Fisher’s exact tests were used to compare determined low bone mineral density between ambulate and non-ambulate groups. Differences were considered significant at *P*<0.05.

## Results

 Thirty boys with DMD were enrolled. The mean age of the patients was 11±3.6 and mean age of symptom onset was 5.1±2 yr. Functional grading of subjects is presented in Table 1.

**Table 1:** Functional levels the study subjects

Grade	Functional level	N (%)
1	Walks unassisted and climbs stairs without assistance	
2	Walks unassisted and climbs stairs with aid of railing	
3	Walks unassisted and climbs stairs slowly with aid of railing (>25s for eight standard steps)	
4	Walks unassisted and rises from chair but cannot climbs stairs	
5	Walks unassisted but cannot rise from chair or climb stairs	
6	Walks only with assistance or walks independently with long leg braces	
7	Walks in long leg braces but requires assistance for balance	
8	Stands in long leg braces but unable to walk even with assistance	
9	Is in a wheelchair	

Patient with scale 1-5 Classified as ambulate (n=21), and with scale 6-9 non-ambulate (n=9). The mean age of loss of ambulation was 9.1±0.9 yr. 

Twenty six patients (86.7%) had osteoporosis according to bone densitometry reports. The prevalence of osteoporosis was not significantly different between two ambulatory and non-ambulatory groups (*P*>0.05). The mean lumbar spine bone density z-score was -1.5±0.22. The mean proximal femur bone density z-score was -3.4±0.25. Bone mineral density in the proximal femur was more profoundly diminished than that in the lumbar spine in both ambulatory and non-ambulatory group (*P*<0.05). 

History of previous fracture was obtained in 7(23.3%) patients. All of the patients were on long-term corticosteroid therapy (17-79 months, mean 48±31 months) and the mean dose of corticosteroid was 0.4±0.15mg/kg/day. Duration of steroid therapy had significant effect on osteoporosis prevalence (*P*=0.03).

Vitamin-D deficiency was detected in 13 patients (Table 2).

**Table 2:** The range of vitamin-D deficiency in 30 DMD patients

Range	Degree of deficiency	Number of patients	Percentage
<10 nmol/l	Severe deficiency	2	6.7
10-20 nmol/l	Deficiency	8	26.7
20-30 nmol/l	Insufficiency	3	10
>30 nmol/l	Sufficient or normal	17	56.7

 Serum vitamin-D level was more profoundly diminished in non-ambulates (88%) comparing ambulates (23%) (*P*=0.008).There was no statistically significant correlation between serum level of vitamin-D and bone density (*P*=0.11).

 The mean dietary calcium intake of patients was 1100±500, 18 patients (60%) had calcium dietary lower than 1200 mg/d and in all patients serum calcium level was within normal range mean=9.7±0.3. Dietary intake of the vitamin in all patients was lower than 15 µg/day. Serum phosphorus levels were within normal limits in all patients (normal range 4-6). PTH level was 40±25 (normal range15-65). Main characteristics of the two groups of patients (ambulate and non-ambulate were compared on Table 3.

**Table 3:** Main characteristics of the two groups of patients

Groups	Ambulate(21)	Non-ambulate(9)	*P*-value
Characteristic			
Osteoporosis	18/21(85%)	8/9(88%)	1
Bone density(g/cm2) (Lumbar spine)	-1.5±0.24	-1.4±0.27	0.7
Bone density(g/cm2) (proximal femur)	-3.4±0.2	-3.4±0.3	0.48
Vit-D deficiency	5/21(23%)	8/9(88%)	0.008

## Discussion

 The problem of deteriorating bone health has been recognized for a long time in patients with DMD but it is only now becoming a topic of research. Two international workshops were organized in 2004 to address the growing treatment of low BMD in patients with DMD. There are several mechanisms involved in poor bone health. Reduced muscle tension on bone and consequent loss of muscular strength is likely significant, however there are many other potential mechanisms, involving chronic inflammation that is a characteristic of DMD wherein muscles lack dystrophin, activation of pathways affecting osteoclastogenesis, calcium homeostasis alteration, long-term steroid therapy as well as changes in vitamin D status (18, 19).

 Approximately 87% of patients with DMD from our study presented low bone mineral density; corroborating other DMD studies that reported decreases in BMD. In a study using Z-score as a measure, eight out of ten of DMD non- steroid treated patients had osteoporosis in the proximal femur (20). Twenty two DMD non- steroid treated patients presented lower BMD before the loss of ambulation and it was correlated with muscular weakness (21). Binachi et al. studied 32 patients with DMD and 22 of them were treated with a long-term steroid, ten cases received no steroids, all of the patients had bone mineral density lower (z-score) than normal for their age and even lower in the steroid treated patients. There was a significant decrease in their spinal bone mineral density and low 25-hydroxyvitamin D levels; also, there was an increased bone turnover markers especially in the steroid-treated group (22). Low bone mineral density has also been reported for steroid treated and non- steroid treated DMD patients in other studies (23).

 Larson et al. investigated fracture prevalence and osteoporosis contributors in 41 patients with DMD. They showed that 18(44%) from 41 patients had sustained at least one fracture, and demonstrated that lumbar spine bone density was affected by the loss of ambulation in contrast to proximal femur that was markedly diminished even in ambulatory phase. In our study the mean bone density in the proximal femur is more profoundly diminished than that in the lumbar spine in both ambulated and non-ambulated patients but in contrast to Larson study osteoporosis severity was not affected by muscle function (24).

 Although corticosteroids slow the progression of the disease, long-term treatment results in osteoporosis, though trabecular bone demineralization and increasing incidence of bone fractures. The possible adverse side effects of corticosteroids therapy directly on bone are difficult to separate from those which may be indirectly beneficial to muscular strength. Indeed BMD appears to be diminished at a very early age regardless of steroid use (25).

An estimated 20%-25% of boys with DMD experience a long bone fracture. In UK, 158 patients with DMD presented fracture prevalence of 20.9%. The use of steroid was not shown to increase the risk of fracture in this investigation (11). History of previous fracture in our study was obtained out of 7(23.3%) patients. The mean duration of corticosteroid therapy was 4±2.6 yr in our study having statistically significant effect on osteoporosis prevalence (*P*=0.03). 

 Diet is extremely important when considering calcium and vitamin -D status in children. Sunshine exposure is another important source of this vitamin. Children with DMD may spend less time exposed to sunshine than other children resulting in lower vitamin -D synthesis and are consequently dependent on dietary intake and supplementation. The maximum net calcium balanced is achieved with dietary intake of 1200-1500 mg/d and vitamin -D intake of 15 μg/d (26). In the present study, 60% of patients had calcium dietary intake lower than 1200 mg/d and all of the patients had vitamin -D dietary intake lower than 10 μg/d.

 In the present study, 43.3% had inadequate vitamin D level and 6.7% showed severe vitamin D deficiency. Serum vitamin -D level was more profoundly diminished in non-ambulates group. In a study, with 24 DMD patients and 24 age-matched healthy boys the PTH levels were within normal range for all subjects. Significantly low levels of 25(OH) D were found in patients group compared with the control group (27). 75% of 117 patient with DMD had inadequate vitamin D level and 14% had severe deficiency (28). 

 Bianchi et al. published a follow-up study of 33 children with DMD being treated with a fixed dose of prednisone. Patients were observed for the first year and were subsequently treated with vitamin D3 plus an adjustment of dietary calcium for two additional years. During the year of observation, bone mineral content and BMD decreased in all patients. At the end of therapy there was a significant increase in bone mineral content and BMD in 65 % of cases (22). 

These results raise important components about the bone disorder in patients with DMD. Components of osteomalacia are suggested by observed improvement of low bone mineral content and BMD with vitamin D repletion. 

However, the particular part of osteoporosis have been discovered but the full correction using vitamin D and calcium nutritional status should be studied more in future research. 

 The bone related disease in DMD not only are due to the osteoporotic nature of this disease but also it might be found in other disease such as osteomalacia. In most of reported cases a mixture of these two illness were presented simultaneously. This adds complexity to the choice of possible therapeutic approaches and emphasizes the importance of achieving a better understanding of the causes of the DMD bone loss in DMD patients (29).

As for limitation of our study, urinary calcium and phosphorus excretion were not measured also evaluation of bone fracture was not surveyed radiologically and only history of fractures was sought. Due to ethical point we could not design case – control study. 

**In conclusion, **there are a higher frequency of the vitamin D deficiency and osteoporosis in DMD patients although osteoporosis severity was not affected by muscle function and ambulation. So it seems that vitamin D supplementation can improve vitamin D status and osteoporosis in these patients, especially in non-ambulates.

## Conflict of interest

The authors declare that there is no conflict of interests.
